# Deep Imaging: How Much of the Proteome Does Current Top-Down Technology Already Resolve?

**DOI:** 10.1371/journal.pone.0086058

**Published:** 2014-01-28

**Authors:** Elise P. Wright, Kali A. G. Prasad, Matthew P. Padula, Jens R. Coorssen

**Affiliations:** 1 Molecular Physiology, and the UWS Molecular Medicine Research Group, School of Medicine, University of Western Sydney, Campbelltown, NSW, Australia; 2 Proteomics Core Facility, Faculty of Science, University of Technology, Sydney, NSW, Australia; Aligarh Muslim University, India

## Abstract

Effective proteome analyses are based on interplay between resolution and detection. It had been claimed that resolution was the main factor limiting the use of two-dimensional gel electrophoresis. Improved protein detection now indicates that this is unlikely to be the case. Using a highly refined protocol, the rat brain proteome was extracted, resolved, and detected. In order to overcome the stain saturation threshold, high abundance protein species were excised from the gel following standard imaging. Gels were then imaged again using longer exposure times, enabling detection of lower abundance, less intensely stained protein species. This resulted in a significant enhancement in the detection of resolved proteins, and a slightly modified digestion protocol enabled effective identification by standard mass spectrometric methods. The data indicate that the resolution required for comprehensive proteome analyses is already available, can assess multiple samples in parallel, and preserve critical information concerning post-translational modifications. Further optimization of staining and detection methods promises additional improvements to this economical, widely accessible and effective top-down approach to proteome analysis.

## Introduction

The main obstacle to effective and comprehensive proteome analysis has ostensibly been resolution. A variety of methods have been investigated in order to resolve ever smaller quantities of protein and detect them quantitatively [1]–[4]. One of the original and most powerful methods has been two-dimensional gel electrophoresis (2DE) [5]. Not only does this yield a position in a gel indicating isoelectric point (pI) and molecular weight, it does so with high reproducibility, and also resolves protein variants including isoforms and post-translationally modified forms (i.e. protein species [6]). While much dogma was associated with this method for a number of years, many of the suggested resolution issues have been addressed, enabling the full spectrum of proteins to be resolved by a refined, standardized protocol for sample preparation and 2DE [7]–[8]. This was largely achieved through the introduction of commercial immobilized pH gradient (IPG) strips [9]–[11], fine tuning of buffer, reducing and detergent components, and the use of fractionation to improve proteome coverage [8], [12]–[13].

In conjunction with these methodological improvements, advances in protein detection have also occurred [14]–[16]; among the most sensitive reagents currently available is the fluorescent stain, SYPRO Ruby (SR). However, there are drawbacks to using such high sensitivity fluorescent reagents to detect proteins. In complex proteome samples with varied protein concentrations, the fluorescence from hyper-abundant proteins rapidly saturates, markedly limiting the total exposure time possible during imaging. As a result, proteins of low abundance are not exposed to enough excitation to yield a measurable signal, and these are thus effectively ‘masked’ by saturation and remain undetected [17]. Thus, removal of higher abundance proteins enables those of lower copy number to be quantitatively assessed [18]–[19]. Such removal of high abundance proteins has previously been attempted by means of pre-fractionation depletion [20]. However, such approaches are costly and give rise to issues of reproducibility, specificity, and quantitative analysis [19]; these are crucial matters in terms of reliable, quantitative proteome analyses, particularly with regard to genuinely understanding physiological functions, molecular mechanisms, and disease states.

Here, we capitalize on the resolution afforded by a refined 2DE protocol and sensitive protein detection using an established fluorescent stain, to test whether significantly more proteins are detectable following excision of hyper-abundant spots. Thus, how much of the proteome is resolved using a standardized 2DE protocol? By now addressing another issue concerning protein detection, we visualize protein species of low abundance in a genuine, well established top-down analytical format, thereby also avoiding the use of multi-step depletion methods which affect sample integrity; in a sense this is an alternate and complementary approach to our original introduction of post-fractionation and third dimension resolution [21]. Thus, excision of highly abundant protein spots followed by another imaging of the gel resulted in the detection of significantly more protein species. Such a deep-imaging approach reaffirms the very high resolution of 2DE as a top-down analytical approach for quantitative proteomic analyses.

## Methods

### Ethics Statement

Rats were originally obtained as breeding pairs under the UWS School of Medicine Animal Facility Rodent Breeding Program with the approval of the UWS Animal Ethics Committee (Approval number: A9710). The donated rat brain tissue used here was obtained from old breeding pairs that had had their fifth litter and were due to be culled (Section 1.26). The method of sacrifice was carbon dioxide asphyxiation. All animals were handled in strict accordance with the UWS Animal Ethics Committee guidelines.

### Chemicals

All materials were of electrophoresis grade or higher and were supplied by Amresco (Solon, OH). SR gel stain and 7 cm 3–10 non linear immobilized pH gradient (IPG) strips were purchased from Bio-Rad (Hercules, CA).

### Sample preparation

Triplicate rat brains were pulverized using automated frozen disruption, and the powdered tissue fractionated as described previously [7]–[8], [12], [21]–[22]. Briefly, the powdered rat brain tissue was lysed in HEPES, neutralized in phosphate buffered saline, and the soluble and membrane protein fractions separated by ultracentrifugation. As previously described, both fractions were then solubilized in 2DE sample buffer containing 8 M urea, 2 M thiourea, and 4% (w/v) 3-[(3-cholamidopropyl)dimethylammonio]-1-propanesulfonate (CHAPS) [7]–[22].

### IPG strip rehydration and 2DE

Reduction and alkylation steps were carried out as described previously [7]–[8], [12], [21]–[22]. Rehydration of IPG strips was for 16 h at room temperature (RT) followed by isoelectric focusing (IEF) at 17°C in the Protean IEF Cell (BioRad). Samples were desalted at 250 V for 15 min, and the voltage was then ramped to 4 000 V over 2 h with wick changes every 30 min. The IPG strips were then focused for 37 500 Vh, and briefly held at 500 V until their application to SDS-PAGE gels for the second dimension of resolution. Samples were resolved in a mini-gel format (10% T, 3.6% C) cast with a stacking gel (5% T, 3.6% C). Equilibration of the IPG strips and SDS-PAGE were carried out as described previously [7]–[8], [12], [21]–[22]. Following protein resolution, each gel was incubated in 50 mL of fixative solution (10% (v/v) methanol, 7% (v/v) acetic acid) on a rocker at 60 rpm for 1 h at RT; each was then washed in 50 mL of double distilled H_2_O (ddH_2_O) and incubated on a rocker for 3×20 min at RT. Staining was by overnight incubation on a rocker in the dark with 40 mL of SR per gel.

### Detection, spot excision, and deep imaging

High sensitivity imaging was accomplished with the LAS-4000 (FujiFilm, Japan) using standard sub-saturation exposure [8], [12], [21]–[22]. Images were then digitally cropped using Multigauge® to exclude dye-front and molecular weight (MW) marker bands. High abundance protein spots of near-saturating signal strength were excised manually. Following excision, the gels were imaged again using the standard approach; this enabled a trebling of the original exposure times from 1 s to 3 s. Although repeated exposure can result in photobleaching [23]–[24], the short exposure times used here (i.e. 1–3 s) were well below concern, and gels were protected from ambient light throughout the analyses.

### Image analysis

Delta 2D (Decodon, Germany) software was used for quantitative image analysis. Gel images were submitted for automated protein spot detection and individual protein spot totals were averaged across replicates. Results were reported as mean ± SEM. Statistical analysis via t-test was carried out using GraphPad Prism®. Artefacts occasionally detected around cut edges of excised spots were excluded from analyses. Delta 2D makes use of a ‘fusion function’ that enables the creation of an average composite gel from replicate gel images; the ‘Average’ algorithm in this function ensures that only spots that are reproducible across all gel replicates are present on a fused composite image. Average fused gel images were thus also created for each experimental condition and protein spot numbers quantified. The spot morphology of the newly detected spots was examined in detail to confirm that the signal was characteristic of a protein spot [25]. Needle-shaped signal spikes characteristic of imaging artefacts were removed from analysis.

### Mass Spectrometry Analysis

A selection of protein spots that were newly detected following deep imaging were identified using liquid chromatography tandem mass spectrometry (LC/MS/MS). Peptides were prepared for MS analysis as described previously [16] with some modifications. Briefly, excised gel spots were rehydrated with 20 µL of 100 mM NH_4_HCO_3_ pH 9 containing 25% of the standard trypsin content (i.e. ∼3 ng/µL). Gel pieces were incubated for 10 min on ice followed by 12 h at RT. The tubes were then sonicated for 30 min and the supernatant removed to a fresh tube. Ammonium bicarbonate (concentration: 50 mM; volume: 30 µL) was added to the hydrated gel pieces and sonication was repeated. Supernatants were pooled and concentrated by speedy vac. Data analysis was carried out as described with the following modifications [16]: variable modifications also included carbamidomethyl. Protein identification was determined based on the number of identifying peptides, the sequence coverage and the significance of the *p*-value. The spectra of proteins identified by a single significant peptide were assessed and annotated but are reported only in Supplementary data (Table S1).

## Results

**Figure 1:**
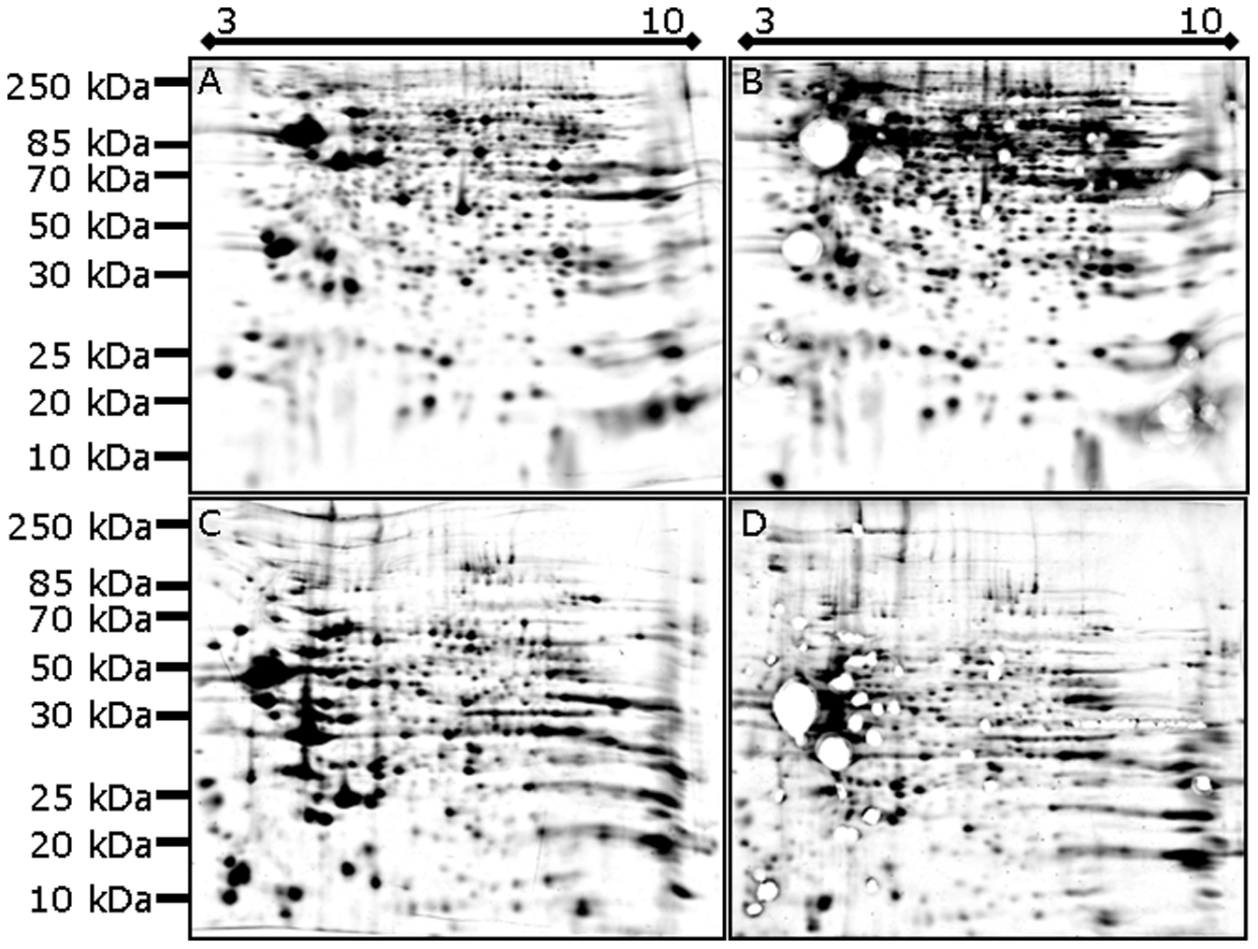
Fused (i.e. average) gel images of resolved soluble (A, B) and membrane (C, D) proteomes from rat brain both before (A, C) and after excision of high abundance proteins (i.e. near-saturating spots; B, D). Following electrophoresis, fixing and staining, gels were imaged for the longest exposure time that produced sub-saturation signal. High abundance spots were then excised from the gel to enable extended imaging exposure time while still yielding sub-saturation signal.

Rat brain soluble and membrane protein fractions were isolated, resolved using 2DE, and stained with SR according to established protocols [7]–[8], [12], [21]–[22]. Following standard imaging, high abundance protein spots were excised from each gel (i.e. reproducibly for replicate gels); protein spot totals were assessed both before and after excision. In the initial assessment, automated image analysis indicated 972±46 resolved soluble protein species (Table 1; Figure 1A). Following excision of 57 high abundance spots, 1231±83 resolved protein species were detected. Thus, on average, 259 resolved protein species (*p* = 0.0213) were initially below the detection limit but could be detected by a single round of deeper imaging (Table 1; Figure 1B). Similar improvements in detection were observed in the analysis of the membrane proteome; image analysis indicated 832±40 and 1144±56 protein species (*p* = 0.004) detected in the pre- and post excision gels, respectively (Table 1). Therefore, the excision of 61 high abundance spots resulted in the detection of an additional 312 membrane protein species (Figure 1C & D). Thus, including excised spots, conservatively, ∼1288 and ∼1205 protein species were resolved and detected in the soluble and membrane proteomes, respectively. This amounts to a total detection of almost 2500 protein species from the mouse brain proteome, *using mini-gels*. This is clearly a minimal estimate of all the resolved/resolvable species [21], even in such small samples [8], [21]. Furthermore, spots revealed by deep imaging that were submitted for sequencing were confirmed as protein rather than staining or imaging artefacts. While these protein species were among the least abundant present on the gel, coverage ranged from 4–47% with multiple significant peptides (*p*<0.05) (Table 2). An ‘averaging’ algorithm (Delta2D) was used to compile fused images that were also submitted for automated spot detection; as expected, by assessing only spots 100% reproducibly detected across replicate gels, this analysis yielded lower total spot counts. On average, 74.5% of protein species detected on the replicate membrane gels were reproducibly detected; 641 protein species were detected on the fused average initial image and 870 protein species on the fused average post-excision image (Table 1). The reproducible proportion was 71.5% in the soluble proteome, represented by 686 and 941 protein species detected in initial and post-excision gel images respectively.

**Table 1:** Spot counts for initial and excised gels, and the total protein counts including excised spots.

	MEMBRANE	SOLUBLE
Initial gel imaging (1 s)	832±40 (n = 5)	972±46 (n = 6)
Total spots excised	61	57
Post-excision gel imaging (3 s)	1144±56 (n = 5)	1231±83 (n = 6)
Post-excision gel spot quantification+total spots excised	1205±58†	1288±74†
Initial average gel (i.e. 100% reproducible spots)	641	686
Post-excision average gel	870	941

Values given are mean ± SEM for total spot counts; average gel counts refer only to spots that were reproducibly detected across all replicate gels and therefore have no error associated with them.

† Error = average percentage error of initial and post excision protein spot counts applied to the sum of the total spots excised and the post excision protein spot totals.

**Table 2:** Protein species identified by LC/MS/MS.

Mascot ID	Gene	Score	Theoretical		Observed		Coverage (%)	Peptides	E-value
			Mass (kDa)	pI	Mass (kDa)	pI			
Q62703	Rcn2 Reticulocalbin-2	192	37.4	4.27	59.5	4.2	13	R.VIDFDENTALDDTEEESFR.Q	4.30E-03
								K.LSEEEILENQDLFLTSEATDYGR.Q	4.80E-07
P47727	Cbr1 Carbonyl reductase [NADPH] 1	168	30.6	8.22	21.0	7.9	9	K.QLQTEGLSPR.F	2.80E-03
								R.SETITEEELVGLMNK.F+Oxidation (M)	5.00E-05
O08838	Amph Amphiphysin	393	74.8	4.57	67.5	4.4	15	K.ADETKDEQFEEYVQNFK.R	2.6
								R.KLVDYDSAR.H	520
								R.RVGFYVNTFK.N	47
								K.AFSIQGAPSDSGPLR.I	8.10E-03
								K.IDVESTELASSESPQAAELEAGAPQEK.V	4.00E-04
								K.VETLHDFEAANSDELTLQR.G	5.70E-06
								K.GLFPENFTR.H	30
B1WC34	Prkcsh Protein Prkcsh	231	59.2	4.41	67.5	4.4	4	K.EKESLQQLAEVTR.E	5.50E-04
								K.SLEDQVETLR.T	1.60E-02
P23565	Ina Alpha-internexin	1510	56.1	5.2	36.6	4.7	43	R.SNVASTAACSSASSLGLGLAYR.R+Propionamide (C)	7.00E-07
								R.LPASDGLDLSQAAAR.T	5.20E-05
								R.TNEKEQLQGLNDR.F	1.70E-06
								R.FAVFIEK.V	4.6
								R.ALEAELAALR.Q	7.10E-03
								R.VGELFQR.E	2.80E-02
								R.AQLEEASSAR.A	6.8
								R.AQALLERDGLAEEVQR.L	2.20E-03
								R.DVDGATLAR.L	290
								K.FANLNEQAAR.S	1.7
								R.TIEIEGLR.G	4.4
								R.QILELEER.H	1400
								R.HSAEVAGYQDSIGQLESDLR.N	1.10E-06
								R.HLREYQDLLNVK.M	0.33
								K.MALDIEIAAYRK.L+Oxidation (M)	4.80E-03
								K.VGESFEETLEETVVSTK.K	4.10E-06
								K.STIEEITTSSSQK.M	2.50E-03
P63018	Hspa8 Heat shock cognate 71 kDa protein	1019	70.8	5.37	36.6	4.7	24	K.VEIIANDQGNR.T	0.15
								R.TTPSYVAFTDTER.L	0.016
								K.NQVAMNPTNTVFDAKR.L+Oxidation (M)	3.30E-07
								K.SFYPEEVSSMVLTK.M+Oxidation (M)	3.00E-02
								R.IINEPTAAAIAYGLDKK.V	1.40E-03
								K.STAGDTHLGGEDFDNR.M	1.50E-03
								R.MVNHFIAEFK.R+Oxidation (M)	0.25
								R.FEELNADLFR.G	1.90E-03
								K.SQIHDIVLVGGSTR.I	8.50E-03
								K.LLQDFFNGKELNK.S	74
								K.NSLESYAFNMK.A+Oxidation (M)	0.11
								K.VCNPIITK.L+Propionamide (C)	52
P63259	Actg1 Actin, cytoplasmic 2	849	41.8	5.31	36.6	4.7	47	K.AGFAGDDAPR.A	6.6
								R.AVFPSIVGRPR.H	4.3
								R.VAPEEHPVLLTEAPLNPK.A	1.60E-02
								R.TTGIVMDSGDGVTHTVPIYEGYALPHAIL R.L+Oxidation (M)	0.19
								R.LDLAGRDLTDYLMK.I+Oxidation (M)	2.8
								K.LCYVALDFEQEMATAASSSSLEK.S+Oxidation (M); Propionamide (C)	3.90E-06
								K.SYELPDGQVITIGNER.F	3.20E-04
								K.DLYANTVLSGGTTMYPGIADR.M+Oxidation (M)	2.60E-09
								R.MQKEITALAPSTMK.I+2 Oxidation (M)	3.80E-04
								K.EITALAPSTMK.I+Oxidation (M)	2.6
								K.IKIIAPPER.K	7
								K.QEYDESGPSIVHR.K	7.10E-02
P68370	Tuba1a Tubulin alpha-1A chain	323	50.1	4.94	36.6	4.7	20	K.TIGGGDDSFNTFFSETGAGK.H	1.60E-04
								R.AVFVDLEPTVIDEVR.T	6.70E-03
								R.NLDIERPTYTNLNR.L	26
								R.IHFPLATYAPVISAEK.A	0.15
								K.DVNAAIATIK.T	110
								K.VGINYQPPTVVPGGDLAK.V	22
Q66HF1	NADH- ubiquinone oxidoreductase 75 kDa subunit, mitochondrial	273	79.4	5.65	36.6	4.7	8	R.FASEIAGVDDLGTTGR.G	6.40E-05
								R.VAGMLQSFEGK.A+Oxidation (M)	4.30E-03
								R.FEAPLFNAR.I	290
								K.KPMVVLGSSALQR.D+Oxidation (M)	25
								K.VAVTPPGLAR.E	0.25
G3V7U4	Lmnb1 Lamin-B1	267	66.6	5.11	36.6	4.7	8	R.ASAPATPLSPTR.L	0.19
								K.DAALATALGDKK.S	7.50E-03
								R.IESLSSQLSNLQK.E	8.60E-04
								K.LLEGEEERLK.L	3.50E-02
P63039	Hspd1 60 kDa heat	231	60.9	5.91	36.6	4.7	8	K.LSDGVAVLK.V	0.85
	shock protein,							R.AAVEEGIVLGGGCALLR.C+Propionamide (C)	4.9
	mitochondrial							K.IGIEIIKR.A	4.90E-03
								K.NAGVEGSLIVEK.I	2.70E-03
P60711	Actb Actin,	204	41.7	5.29	35.5	5.3	14	R.AVFPSIVGRPR.H	6200
	cytoplasmic 1							R.VAPEEHPVLLTEAPLNPK.A	3.30E-03
								R.VAPEEHPVLLTEAPLNPK.A	5700
								K.SYELPDGQVITIGNER.F	0.000061
								K.EITALAPSTMK.I+Oxidation (M)	290

A subset of protein species specifically detected by deep imaging were prepared for identification by MS and analysed as described previously [16]. All identified proteins were from the *Rattus norvegicus* species.

## Discussion

The issue of proteome coverage using available analytical methods is well established. For decades, stain developments have driven routine detection of diminishing quantities of protein [26]. This has included methodological improvements and the application of novel compounds. Consider the vastly improved detection obtained with the introduction of a colloidal formulation of Coomassie Brilliant Blue [14], [27]; yet the same resolution and detection methods were still being used - proteins were always being resolved, but were simply below the threshold of the contemporary detection methods. This was even clearer when comparing the detection sensitivity of different stains on 2DE gels; when resolved using the same method as in the current study, more than twice as many protein species were detected when the same gels were imaged using an infrared protocol [22]. The proteins had already been successfully resolved but they remained undetectable without improvements in detection.

Here we tested a deep imaging approach: could resolved proteins of even lower abundance be detected following the excision of saturating protein spots? By thus extending the saturation threshold (i.e. essentially an extension of our original 3D post-fractionation analytical approach [21]), it was possible to excite less intensely stained proteins for longer, bringing them into the range of detection. The data indicate that the resolving power of 2DE is indeed equal to the task of analyzing complex proteomes, particularly with regard to potential biomarkers. It also emphasizes that the main obstacle to effective and comprehensive proteome analysis is likely detection rather than resolution.

Taking the excised spots into account, a single round of deeper imaging detected ∼33% and 45% more membrane and soluble protein species, respectively. The excision of hyper-abundant spots is an important factor in this increased detection. While signal can be raised across a gel by merging multiple exposures, it is impossible to avoid the signal bleed that results from this process. By removing the source of the saturating signal, the basic topography of the gel image is undisturbed; tight clusters of spots or those near a larger spot can still be distinguished as single entities. Furthermore, this approach does not affect the comparative image analysis or the conclusions that can be drawn from these gel images. Protein quantification between conditions continues to be relative to control gel images. Thus, standard, relative quantitative analyses can still be carried out using spots detected in either the pre- or the post-excision gel images.

LC/MS/MS was carried out to confirm that the newly detected spots were indeed protein species rather than staining or imaging artefacts. Not surprisingly, as has been the case throughout the last 40–50 years of stain development and enhanced in-gel protein detection, newly detected spots proved to be proteins/protein species, and some are recognised to be of low abundance. For example, 14-3-3 protein zeta/delta (P63213), Carbonyl reductase (NADPH)1 (P47727), and reticulocalbin-2 (Q62703) were also identified in a study that sought to enrich low abundance proteins [28]. The regulatory protein that is enriched in nerve terminals, amphiphysin (O08838), was also present amongst the proteins identified here and has also been noted as being of low abundance [29], [30]. That these protein species are being detected here in a total brain extract further confirms that deep imaging offers an excellent opportunity to quantify difficult to detect protein species as part of more comprehensive proteome analyses.

Considering their near-saturation abundance, most, if not all of the excised spots likely contained multiple resolved protein species; this has been established previously by quantitative analysis [21]. Furthermore, in terms of consistent and reproducible proteome analyses, only ∼73% of protein spots were 100% reproducible across all replicates. This suggests that ∼30% of protein species identified after excision were on the very cusp of lower detection limits. As this analysis only represents a single round of abundant protein excision it is likely that removal of additional protein spots would further substantially increase the number of detectable protein species. Thus, the assessment of total resolved/resolvable protein species on these 2D gels is undoubtedly low [21]. A conservative estimate based on previous work would include: (i) ∼4–7 co-migrating proteins per hyper-abundant protein spot; (ii) ∼40–50 additional proteins at pI extremes; (iii) ∼60 proteins co-migrating with the dye front; (iv) all of the protein spots detected following saturating protein spot excision; (v) and all of the excised spots. All told, this suggests a total of ∼2800–3000 protein spots. This range of resolved and detected protein species is minimally comparable to claims of proteome coverage made from routine shotgun MS analyses [31]; here we also take reproducibility of protein spot detection into account. We can only speculate as to the substantial potential improvements these straightforward assessments will make considering the proteome resolution and coverage already achieved in the standardized very large gel format used by Klose and colleagues [32].

Considering the extent of proteome resolution shown here, the ability to resolve multiple samples in parallel, and with its routine information on isoelectric point, approximate MW, abundance, isoforms and post-translational modifications, the results here move 2DE well away from the antiquated dogma that sometimes surrounds it in the literature [33]. Clearly the data here again confirm 2DE as a genuine, quantitative, state-of-the-art top-down approach to proteome analyses. What other method is capable of such high resolution, and of resolving multiple samples in parallel, while concurrently providing physicochemical information [33]? At present, it would seem only 2DE yields quantitative data, and combined with MS, identifies proteins based on high sequence coverage [16]. Thus, it seems reasonable to conclude that many of the tools needed to address fundamental biological questions are already in our hands provided we apply them rigorously, and always with an eye to further improvements in technology and methodology. This need not be expensive or require the highest-end of instrumentation, but rather it is a matter of the rigor with which techniques are applied and the best possible data obtained from them. There is simply no longer room for the sort of dogma that seems to have tainted proteomics for some time now.

## Supporting Information

Table S1 **Protein species identified by LC/MS/MS.** A subset of protein species specifically detected by deep imaging were prepared for identification by MS and analysed as described previously [16]. These proteins were identified by a single significant peptide. All identified proteins were from the *Rattus norvegicus* species.
(DOCX)
