# Supplementary material for: Deep Imaging: How Much of the Proteome Does Current Top-Down Technology Already Resolve?
Source: PLoS One. 2014 Jan 28;9(1):e86058. doi: 10.1371/journal.pone.0086058 (PMC3904854; doi:10.1371/journal.pone.0086058)
Supplement: Table S1 — Protein species identified by LC/MS/MS. A subset of protein species specifically detected by deep imaging were prepared for identification by MS and analysed as described previously [16]. These proteins were identified by a single significant peptide. All identified proteins were from the Rattus norvegicus species. (DOCX) [file pone.0086058.s001.docx]

| **Mascot ID** | **Gene** | **Score** | **Theoretical** | | **Observed** | | **Coverage (%)** | **Peptides** | **E-value** |
| --- | --- | --- | --- | --- | --- | --- | --- | --- | --- |
|  |  |  | **Mass** | **pI** | **Mass** | **pI** |  |  |  |
| B2RYG6 | Otub1 Ubiquitin thioesterase OTUB1 | 119 | 31.3 | 4.85 | 34.4 | 4.5 | 9 | R.IQQEIAVQNPLVSER.L | 2.90E-04 |
| P63102 | Ywhaz 14-3-3 protein zeta/delta | 95 | 27.8 | 4.73 | 34.4 | 4.5 | 12 | K.GIVDQSQQAYQEAFEISK.K | 7.10E-03 |
| Q52KS1 | Pfkm 6-phosphofructokinase | 101 | 85.3 | 8.07 | 131.9 | 7.2 | 2 | K.AIAVLTSGGDAQGMNAAVR.A + Oxidation (M) | 7.90E-06 |
| M0RCH6 | LOC100359642 Protein LOC100359642 | 85 | 22.4 | 4.72 | 34.4 | 4.5 | 7 | K.QLAQIDGTLSTIEFQR.E | 3.60E-04 |
| Q32Q65 | FGB Fibrinogen beta chain | 78 | 55.9 | 8.54 | 18.5 | 4.4 | 2 | S.QGVNDNEEGFFSAR.G + Deamidated (NQ) | 7.10E-03 |
| P09951 | Syn1 Synapsin-1 | 249 | 73.9 | 9.81 | 51.6 | 8.7 | 10 | K.QTTAAAAATFSEQVGGGSGGAGR.G | 3.90E-06 |
| Q62826 | Hnrnpm Heterogeneous nuclear ribonucleoprotein M | 127 | 73.7 | 8.9 | 51.6 | 8.7 | 4 | R.MGPAMGPALGAGIER.M + 2 Oxidation (M) | 1.20E-03 |
